# Identification and Characterization of the Regions Involved in the Nuclear Translocation of the Heterodimeric Leishmanial DNA Topoisomerase IB

**DOI:** 10.1371/journal.pone.0073565

**Published:** 2013-09-02

**Authors:** Christopher F. Prada, Raquel Álvarez-Velilla, Rosario Díaz-Gozález, Yolanda Pérez-Pertejo, Rafael Balaña-Fouce, Rosa M. Reguera

**Affiliations:** 1 Departamento de Ciencias Biomédicas, Universidad de León, Campus de Vegazana, León, Spain; 2 Instituto de Parasitología y Biomedicina "López-Neyra", Parque Tecnológico de Ciencias de la Salud, Granada, Spain; St. Georges University of London, United Kingdom

## Abstract

*Leishmania donovani*, the causative organism for visceral leishmaniasis, contains a unique heterodimeric DNA-topoisomerase IB (LdTopIB). LdTopIB is a heterodimer made up of a large subunit and a small subunit that must interact with each other to build an active enzyme able to solve the topological tensions on the DNA. As LdTopIB is located within the nucleus, one or more nuclear localization signals (NLS) should exist to ensure its nuclear translocation. In this report three novel NLS have been identified through a sequential deletion study of the genes encoding of both subunits fused to that encoding the green fluorescent protein (GFP). NLS1 is a highly basic sequence of 43 amino acids in the C-terminal extension of the large protomer. We found two well-defined sequences in the small protomer: NLS2 is a 10-amino acid motif located in the N-terminal extension of the protein; NLS3 consists of a complex region of 28 amino acids placed in the vicinity of the catalytic Tyr-222 included at the conserved SKINY signature within the C-terminal. Furthermore, by means of yeast cell viability assays, conducted with several LdTopIB chimeras lacking any of the NLS motives, we have revealed that both subunits are transported independently to the nucleus. There was no evidence of LdTopIB accumulation in mitochondria or association to the kinetoplast DNA network. The results rule out the former hypothesis, which attributes nucleocytoplasmic transport of LdTopIB entirely to the large subunit. The LdTopIB is localized to the nucleus only.

## Introduction

Visceral leishmaniasis is a growing scourge in an increasingly globalized world, where biological and environmental barriers fluctuate constantly. The causative agent, *Leishmania donovani*, has several evolutionary peculiarities that hinder the fight against this disease. These include, the plasticity of its genome [1] or the rapid emergence of drug resistances, between others [2]. Current drugs used against visceral leishmaniasis include old-fashioned pentavalent antimony salts, macrolides, aromatic diamidines and the promising but expensive liposomal amphotericin B [3,4]. However, most of these compounds have plenty of undesirable side effects, such as their high toxicity or the need for tedious and long-term parental treatments [5].

Monomeric eukaryotic type I DNA topoisomerases (TopIB) are nuclear enzymes that catalyze changes in the superhelicity of duplex DNA, thus introducing transient single-stranded breaks in DNA followed by passage and re-joining [6]. This process is known as "controlled rotation" due to the stabilising role of the linker domain [7]. For a long time, these enzymes have been considered as effective drug targets in cancer therapy and due to this, several TopIB inhibitors are currently in clinical use [8]. Unlike the rest of organisms described at present, TopIB from trypanosomatids are heterodimers composed of a large and a small subunit [9,10]. The large LdTopIL protomer is a 73-kDa polypeptide containing a short and non-conserved N-terminal end followed by a putative conserved DNA-interaction region. However, the homology is dramatically lost beyond Lys-471, starting and non-conserved C-extension domain. This in turn, establishes charge interactions with the N-terminal end of the small subunit LdTopIS [11]. This long extension precedes the phylogenetically conserved C-terminal which harbors the phylogenetically conserved catalytic Tyr-222 included within the "SKxxY" signature responsible for the nucleophilic during DNA-cleavage step [12].

Several studies have shown that eukaryotic TopIB have dual, nucleus and mitochondrial compartmentalization [13]. Trypanosomatids are early divergent primitive eukaryotes that contain single modified mitochondria bearing a singular organelle adjacent to the flagellar basal body called a kinetoplast. This organelle contains an intricate network of circular DNA arranged in mini and maxicircles (kinetoplast DNA or kDNA), which have been seen as a potential drug target [14]. Replication of kDNA occurs synchronously with nuclear DNA during cell division. Immunocytochemical localization experiments carried out on trypanosomatids show dual localization of type II DNA topoisomerase (TopII) associated with both genomic DNA in the nucleus and kDNA minicircles in the kinetoplast [15]. Even more controversially, is the role played by type I topoisomerases in these microorganisms. BoseDasgupta and co-workers have reported that *L. donovani* TopIB targets the Universal Minicircle Binding Protein Sequence (UMBPS) in the kinetoplast [16]. However, other researchers point to a TopIA operating enzyme from prokaryotic origin [17]. A simple analysis of the sequences involved in nuclear targeting in *Leishmania* TopIB reveals that all putative NLS are concentrated within C-terminal extension of the large monomer [18]. Owing to this fact, it was proposed that enzyme assembly takes place in the cytosol and then LdTopIL drives the whole enzyme into the nucleus [16].

Only a few reports describe functional NLS in *Leishmania* [19–22]. Due to this, it is not possible to find a common sequence for nuclear transport in these parasites. Both, the nature and length of these sequences are heterogeneous factors. While Kumar and co-workers [19] found a highly basic tetrapeptide at the N-terminal extension of OCR1 protein, another sequence-structure study delimited a NLS region of 60 amino acids in the leishmanial tyrosyl-DNA phosphodiesterase 1 (Tdp1) protein [20].

In the present report we have delineated three novel Nuclear Localization Signals (NLS) by means of a deletion study involving both subunits fused to the Green Fluorescent Protein (GFP). NLS1 is a highly basic 43-amino acid sequence placed at the C-terminal extension of LdTopIL. NLS2 is a 10-amino acid sequence within the N-terminal extension of LdTopIS. Further to this, NLS3 is a more complex region of 28 amino acids in the vicinity of the catalytic tyrosine, which includes the conserved SKINY motif within the C terminus of the small subunit. In addition, we provide evidence that neither LdTopIL, nor LdTopIS fused chimeras are driven to the mitochondria and/or kDNA, overruling the hypothesis of a bi-located TopIB in trypanosomatids. Finally, spot tests conducted with LdTopIB ΔNLS chimeras revealed that both subunits could be transported to the nucleus independently.

## Materials and Methods

### Reagents and culture media

*Pyrococcus woesei* (Pwo) polymerase, DNA modification as well as restriction enzymes were procured from Roche (Basel, Switzerland) and Amersham Biosciences. DNA ligase from T4 bacteriophage was from Stratagene (La Jolla, CA, USA). Cell culture media, chemicals and reagents were purchased from Sigma (St. Louis, MO USA). Primers for PCR amplification were from Sigma Genosys (UK).

### Leishmanial and yeast strains

*L. donovani* LEM75 (Ethiopian) promastigotes were a kind gift from Dr. J.M. Requena (Centro de Biología Molecular "Severo Ochoa", CSIC Madrid, Spain). Promastigotes were routinely cultured in Medium 199 (Sigma Aldrich, St Louis, MO), supplemented with 10% (v/v) heat-inactivated foetal calf serum (FCS) and antibiotics. TopIB-deficient *Saccharomyces cerevisiae* MBY3 strain [MAT α ura3-52 his3Δ200 leu2 Δ1 trp1 Δ63, top1 Δ::TRP1 rad52 Δ::LEU2] for cytotoxic assays [23] was generously gifted by Dr. M.A. Bjornsti (St. Jude’s Hospital, Memphis, TN USA).

### LdTopIB cloning and GFP-TopIB fusion constructs

Heterodimeric wild-type LdTopIB was cloned as described previously [11]. A version of GFP in which the emission spectra had been shifted by a S^65^ to T substitution, cloned in the *Leishmania* expression vector (pXG-GFP+2) was kindly supplied by Dr. Steve Beverley (Dpt. Microbiology, University of Washington at St. Louis, MO, USA). This vector was used to clone in-frame with GFP-open reading frame, several length fragments of both large and small LdTopIB subunits. Different fragments of the C-terminal extension end of LdTopIL and LdTopIS were generated by the Polymerase Chain Reaction (PCR). The sequence of the primers used for *LdTopIL* gene amplification and their positions are listed in Table 1, while primers used for *LdTopIS* gene amplification are listed in Table 2. The PCR reaction contained 20 ng of plasmid *pSK-LdTopIL* or *pSK-LdTopIS* as template, 250 ng of each oligonucleotide, 100 µM dNTPs, 5 µl of 10×*Pwo* buffer and 2.5 units of *Pwo*-polymerase for a total volume of 50 µl. PCR products were cloned in-frame downstream the GFP-ORF at *BamH*1-*Mun*I site of pXG-GFP+2 multiple cloning site. Sequences across cloning sites and all regions generated by PCR were confirmed by sequencing (Instituto de Biotecnología INBIOTEC, León Spain).

**Table 1:** Sequence of the primers used in this study to create the LdTopIL-GFP fusion chimeras.

		
TopL1	FW	CGGGATCCACCATGAAGGTGGAGAATAGCAAGAT
	RV	CAATTGTCACTACACCCTCAAAGCTGCAAG
TopL2	FW	CGGGATCCACCATGAAGGTGGAGAATAGCAAGAT
	RV	CAATTGTCAGTCCCACACGCAGTATCGGT
TopL3	FW	CGGGATCCACCGGGCGTCGCGAGCAGGTG
	RV	CAATTGTCAGATGTGCTCCACACGCAGCG
TopL4	FW	CGGGATCCACCCAGCTCATGCCGGACAACATC
	RV	CAATTGTCAGGCGCCCGACGTGGATTTC
TopL5	FW	CGGGATCCACCAAGAAGGCCGAGTCCGCATC
	RV	CAATTGTCACTACACCCTCAAAGCTGCAAG
TopL6	FW	CGGGATCCACCCAGCTCATGCCGGACAACATC
	RV	CAATTGTCACTTCTTCTTTTTGGCGGCCCTC
TopL7	FW	CGGGATCCACCTCTGCCAAGAAGGGTGGAAAG
	RV	CAATTGTCACTACACCCTCAAAGCTGCAAG
TopL8	FW	CGGGATCCACCGCAGCAAGCAAGTCGTCCAAG
	RV	CAATTGTCACTACACCCTCAAAGCTGCAAG
TopL9	FW	CGGGATCCACCGAGGAGGACGAGGACGAC
	RV	CAATTGTCACTACACCCTCAAAGCTGCAAG
TopL10	FW	AAATCCACGTCGGGCGCCAAAAAGAAGAAGTCTGCC
	RV	GGCAGACTTCTTCTTTTTGGCGCCCGACGTGGATTT
TopL11	FW	GGTGGAAAGGTGTTGAGCGAGGAGGACGAGGACGAC
	RV	GTCGTCCTCGTCCTCCTCGCTCAACACCTTTCCACC

FW: Forward primer; RW: Reverse primer

**Table 2:** Sequence of the primers used in this study to create the LdTopIS-GFP fusion chimeras.


TopS1	FW	CGGGATCCACCATGCAGCCTGTTCAAAGTCCAG
	RV	CAATTGTCAAAAATCGAAGTTCTCGGCAT
TopS2	FW	CGGGATCCACCATGCAGCCTGTTCAAAGTCCAG
	RV	CAATTGTCAAGCCGTCGTTGCAACCGGC
TopS3	FW	CGGGATCCACCGCTCCGCCACCGAAGGTGC
	RV	CAATTGTCAGACGAGGAGGAGCGATCG
TopS4	FW	CGGGATCCACCGCTGAGTCAGTGGTGTCAGG
	RV	CAATTGTCACAGCGTGGGGACTTCCTCC
TopS5	FW	CGGGATCCACCGTGCCTCCGCGTCCTCCG
	RV	CAATTGTCAAAAATCGAAGTTCTCGGCAT
TopS6	FW	CGGGATCCACCATGCAGCCTGTTCAAAGTCCAG
	RV	CAATTGTCAGGAGGACGAGGACGAGCTATC
TopS7	FW	CGGGATCCACCATGCAGCCTGTTCAAAGTCCAG
	RV	CAATTGTCAATCGGACGATGAGCTGGAACTG
TopS8	FW	CGGGATCCACCATGCAGCCTGTTCAAAGTCCAG
	RV	CAATTGTCAGAGCAGTTCCAGCTCATCGTCC
TopS9	FW	CGGGATCCACCATGCAGCCTGTTCAAAGTCCAG
	RV	CAATTGTCACTCGACGCGGCGCACCTTC
TopS10	FW	CGGGATCCACCATGCAGCCTGTTCAAAGTCCAG
	RV	CAATTGTCAGCTCTCGACGCGGCGCACC
TopS11	FW	CGGGATCCACCTCCAGCTCATCGTCCGATAGC
	RV	CAATTGTCACAGCGTGGGGACTTCCTCCTC
TopS12	FW	CGGGATCCACCGTGCCTCCGCGTCCTCCG
	RV	CAATTGTCAGGTGCCGAGCGACACAGCCTT
TopS13	FW	CCGGTTGCAACGACGGCTAGTTCCAGCTCATCGTCC
	RV	GGACGATGAGCTGGAACTAGCCGTCGTTGCAACCGG
TopS14	FW	GCTGTGTCGCTCGGCACCCCCCGTATCATCTGCTCG
	RV	CGAGCAGATGATACGGGGGGTGCCGAGCGACACAGC
TopS15	FW	AAAATCAACTACATCGACCAAGACGTGCCGATCAAC
	RV	GTTGATCGGCACGTCTTGGTCGATGTAGTTGATTTT
TopS16	FW	TGCTCGTGGGCAAAGGCGACCATCCAGAAGAAGTTC
	RV	GAACTTCTTCTGGATGGTCGCCTTTGCCCACGAGCA
TopS17	FW	CGGGATCCACCGTGCCTCCGCGTCCTCCG
	RV	CAATTGTCAGGTTGCAGAGAAGATCTTGTTG
TopS18	FW	CGGGATCCACCATGCAGCCTGTTCAAAGTCCAG
	RV	CAATTGTCAGGTGCCGAGCGACACAGCCTT

FW: Forward primer; RV: Reverse primer

### Bis-cistronic expression of LdTopIB

The different constructs were obtained by cloning the distinct PCR fragments in pBluescript SK(-) vector using current molecular biology techniques. These constructs were subcloned into the bis-cistronic pESC-URA vector. *BamH* I and *Xho*I were the selected restriction sites for the gene encoding the large subunit, whereas *Not*I and *Spe*I were chosen for the small one under control of GAL1 and GAL10 promoters, respectively. The sequence of the primers used for *LdTopIL* and *LdTopIS* genes amplification and their positions are listed in Table 3. Constructs were sequenced in order to confirm that undesirable additional mutations have not been generated into the sequence.

**Table 3:** Sequence of the primers used in this study to create the ΔNLS LdTopIB chimeras.

		
LdTopIL	FW	CGGGATCCATGAAGGTGGAGAATAGC
	RV	CCGCTCGAGCTACACCCTCAAAGCTGC
LdTopIS	FW	ATAAGAATGCGGCCGCATGCAGCCTTTCAAAGTCCT
	RV	GACTAGTGGAGATCAAGTCGCGC
ΔNLS1	FW	CGGGATCCATGAAGGTGGAGAATAGC
	RV	CCGCTCGAGCTAGGCGGCCCTCTTCTTGCCAG
ΔNLS2	FW	CCGGTTGCAACGACGGCTAGTTCCAGCTCATCGTCC
	RV	GGACGATGAGCTGGAACTAGCCGTCGTTGCAACCGG
ΔNLS3	FW	GCTGTGTCGCTCGGCACCCCCCGTATCATCTGCTCG
	RV	CGAGCAGATGATACGGGGGGTGCCGAGCGACACAGC

FW: Forward primer; RV: Reverse primer

### Site-directed mutagenesis of LdTopIB

Internal deletions were developed attending to the QuickChange® method: the genes encoding for both LdTopIS and LdTopIL were cloned in the pBluescript SK(-) vector and then subcloned into the pESC-URA and pXG-GFP+2 vectors.

The sequences of the primers used for this internal mutation are shown in Table 1 and Table 2 for LdTopIL and LdTopIS, respectively. The PCR reaction included 1 µM of each oligonucleotide, 10 µM dNTPs, 20 ng of plasmid pSK-*LdTopIS* or pSK-*LdTopIL* as templates, 5 µl of 10 x *Pwo*-buffer and 2.5 units of *Pwo*-polymerase for a total volume of 50 µl. Reactions were carried out in a Mastercycler gradient thermocycler (Eppendorf®). After PCR, in order to digest the parental DNA template, products were incubated in presence of *Dpn*I, and transformed in DH5α *E. coli*; five clones were sequenced to be sure that site-directed mutation had been introduced accurately. Chimeras were sequenced in order to confirm that undesirable additional mutations have not been included into the sequence.

### Transfection of L. donovani promastigotes

*L. donovani* promastigotes were grown in 199 medium supplemented with 10% (v/v) heat inactivated FCS up to 5x10^6^ cells/well, washed in cold cytomix (120 mM KCl, 0.15 mM CaCl_2_, 10 mM K_2_HPO_4_, 25 mM Hepes pH 7.6, 2 mM EDTA, 5 mM MgCl_2_) and resuspended in the same solution at a density of 1x10^8^ cells/ml. Five hundred-microliter aliquots were electroporated twice with 10 µg of linear GFP-LdTopIB fragments (1.5 kV, 25 µF using a Bio-Rad Gene Pulser II apparatus) in 0.4 cm electrode gap cuvettes, transferred to 10 ml of M199 plus 10% FCS and incubated at 26 ^°^C for 8 h in absence of antibiotics. Cells were spun down, and the pellet resuspended in 100 µl of fresh M199 plus 10% (v/v) FCS and plated on semisolid medium containing 10 µg/ml G418 [24].

### Microscopy analysis and Top-GFP detection

To assess the localization of GFP-fusion proteins in *L. donovani*, promastigotes expressing GFP were collected by low speed centrifugation, washed once and resuspended in PBS at a cell density of 2x10^8^ cells/ml. Total – both nuclear and kinetoplast – DNA was stained by incubation with a 4’,6-diamidino-2-phenylindole (DAPI) solution (146 mM NaCl, 10 mM HCl Tris pH 7.4, 2 mM CaCl_2_, 22 mM MgCl_2_, 50 mg/ml bovine serum albumin and 10 mg/ml DAPI) for 30 min. One hundred and fifty microliters of this suspension was attached to poly-L-lysine coated IBIDI micro-slides (IBIDI GmbH, Martinsried, Germany) after centrifugation at 450 rpm for 5 min. GFP ﬂuorescence was visualized by illumination with UV light and a FITC ﬁlter using a Nikon confocal microscope equipped with a Cooled Coupled Device (CCD) camera.

### Yeast cell viability assays (spot tests)

To analyse the *in vivo* sensitivity of LdTopIB chimeras to camptothecin (CPT), *S. cerevisiae* MBY3 strain was transformed with the wild type protein and ∆NLS-truncated chimeras made onto *LdTopIS* and *LdTopIL* genes driven by GAL1/GAL10 promoters. We used the lithium-acetate yeast-transformation method for this purpose [25]. The bicistronic pESC-URA expression vector carries the URA3 selection marker and was grown by selection in uracil deﬁcient (URA-) media. After an overnight, cultures were adjusted to OD_595_ = 0.3 and serially diluted 10-fold. A drop of 5 microliters was spotted onto selective solid media under two different expression conditions: induced (2% (w/v) galactose) or repressed (2% (w/v) dextrose), in the presence or absence of 15 µM and 30 µM CPT [23]. As control, assay was performed with the pESC-URA expressing vector without any insert (“mock”) under same conditions as the wt-transformed vector. All plates contained 0.4% DMSO.

## Results

### A basic region at the N-terminal extension of LdTopIL constitutes a NLS complex

**Figure 1:**
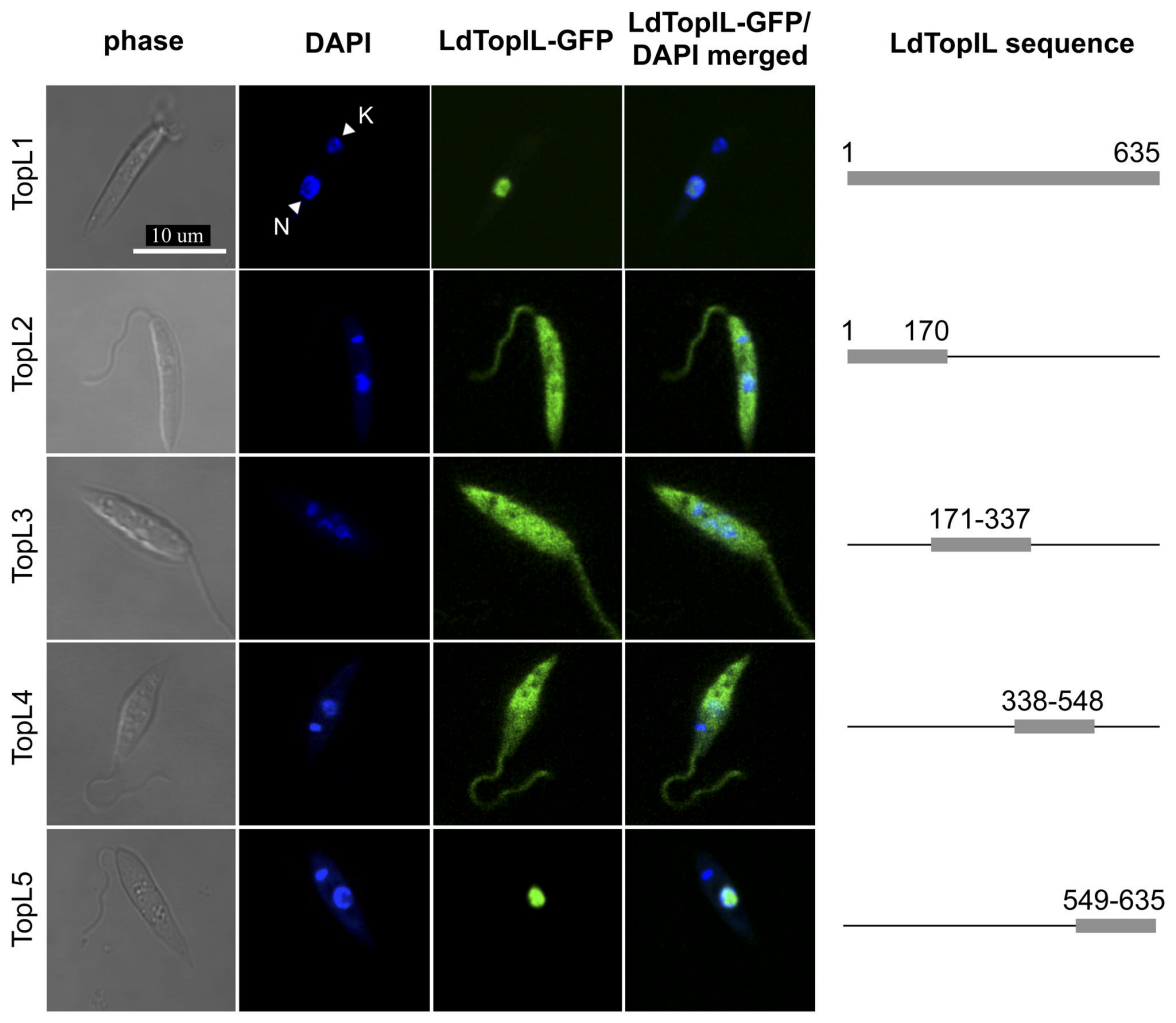
Microscopy confocal analysis of different fragments of the large LdTopIL subunit fused to GFP. Nucleus (N); kDNA (K). Genomic and kDNA staining was carried out with DAPI.

Previous studies have shown that the C-terminal extension of LdTopIL subunit should contain NLS motifs able to target this protein in the nucleus [18]. Towards examining the possible NLS sequences in LdTopIL, we expressed the full-length protein fused to the *Leishmania* expression vector pXG-GFP+2 (GFP was fused at the N-terminal end of the protein). This fusion chimera (TopL1), which involves the entire subunit (aa 1-635), was cloned and transfected into *L. donovani* promastigotes. As expected, TopL1 targeted the nucleus with no cytoplasmic compartmentalization (Figure 1).

To further investigate the sequence that specifically targets LdTopIL subunit to the nucleus we divided the protein into four large fragments and fused them to GFP. The constructs covering the different protein fragments were cloned and transfected. The first fragment, TopL2 (aa 1-170), included the short and non-conserved N-terminal extension, as well as the first 125 amino acids of the core domain. Since NLS of the human TopIB are placed at its N-terminal domain, we expected to find a similar distribution in LdTopIL. Surprisingly, this chimera behaved differently from TopL1, with the protein fluorescence scattering throughout the cytoplasm. As no NLS was found in this region, we prepared TopL3 and TopL4 that ranged from residues 171 to 337 and 338 to 548, respectively. The main feature of TopL3 construct is the presence of the catalytic Arg-314 residue. Meanwhile, TopL4 represents the most conserved region of the protein, harboring the other three residues involved in the catalytic activity; Lys-353, Arg-410 and His-453. The subcellular localization of TopL3 and TopL4 was examined by direct fluorescence of transfected cells. Figure 1 shows that the fluorescence of both chimeras was dispersed in the cytoplasm and as a result, at least a NLS motif should be placed at the C-terminal extension. When the end fragment of the protein (TopL5) was expressed in *L. donovani* promastigotes, the fluorescence was localized in the cell nucleus only [18].

**Figure 2:**
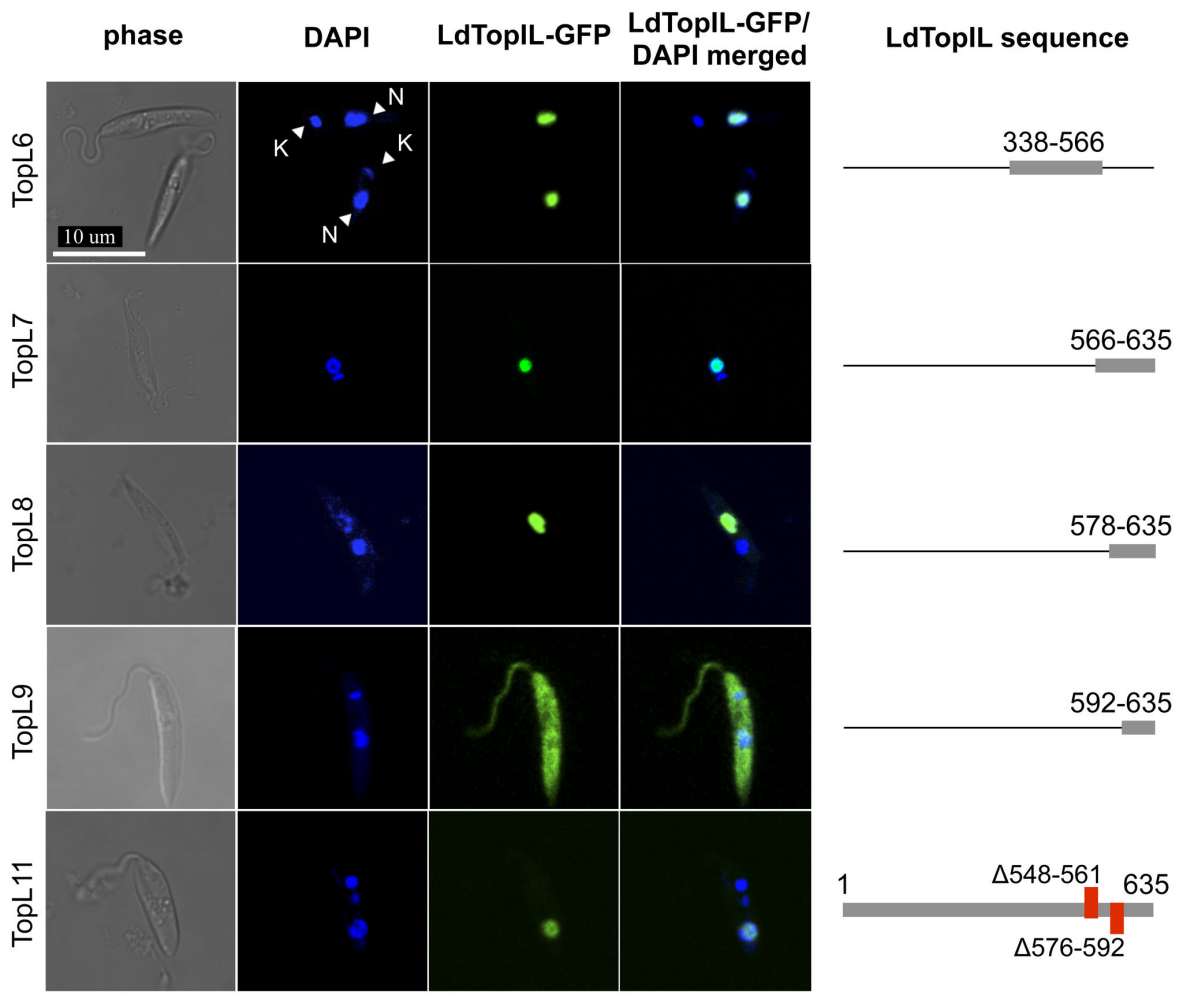
Identification of a NLS (red) in the LdTopIL subunit fused to GFP, by confocal microscopy. Nucleus (N); kDNA (K). Genomic and kDNA staining was carried out with DAPI.

Once the region responsible for the nuclear translocation of LdTopIL was roughly determined, we proceeded to define the NLS with more accuracy through the analysis of the constructs shown in Figure 2. The C-terminal extension harboring the putative NLS was split into two fragments enriched with basic residues, TopL6 and TopL7. TopL6 is an extension of TopL4 containing the unexamined amino acids 548 to 566, which includes eight Lys residues, whereas TopL7 covers the amino-acid sequence from residue 566 to the end of the protein, which also comprises of a cluster of eleven Lys residues at the beginning of the construction. When their subcellular localization was analyzed, both chimeras accumulated in the promastigote nucleus. Since this strategy was unsuccessful in NLS delimitation, we decided to identify the sequence of the C-terminal end, which excludes the nuclear transport of the protein. For this purpose, TopL8 and TopL9 were cloned and fused to the GFP expression vector. While TopL8 comprises of the residues ranging positions 578 to 635, TopL9 starts at residue 592 and also extends to the end of the subunit. As shown in the fourth row of Figure 2, the last 43 amino acids of the LdTopIL are dispensable for its nuclear location, whilst residues 578 to 592 are sufficient for nuclear targeting.

At this end we have successfully identified a highly basic region of 44 amino acids (aa 549-592) able to target the whole subunit to the nucleus. By analyzing these constructions all together we can state that Lys-enriched stretches involving residues 548-566 and 578-592 could constitute a putative NLS. However, the role played by the amino acids between these two regions remained unclear. In order to identify this role, we performed two serial internal deletions removing these nuclear-targeting regions. In the first instance the sequence between residues 548 to 566 was deleted (TopL10), which was used as template for the second internal deletion process removing amino acids 578 to 592 yielding the TopL11 construct. Confocal analysis of this last chimera revealed a clear nuclear localization of green fluorescence, thus suggesting that this 14-amino acid sequence (aa 562-575) acts as a potential NLS. Consequently, one would think that LdTopIL contains three NLS regions. Nonetheless, the sequence continuity as well as the high density of basic residues makes us think that it may be a NLS complex (NLS1; aa 549-592), in which several stretches can assume the role of NLS when any of them is removed. This makes it impossible to further delineate a smaller sequence responsible for this nuclear-transport process.

### Delimitation of two novel NLS involved in the nuclear transport of LdTopIS

**Figure 3:**
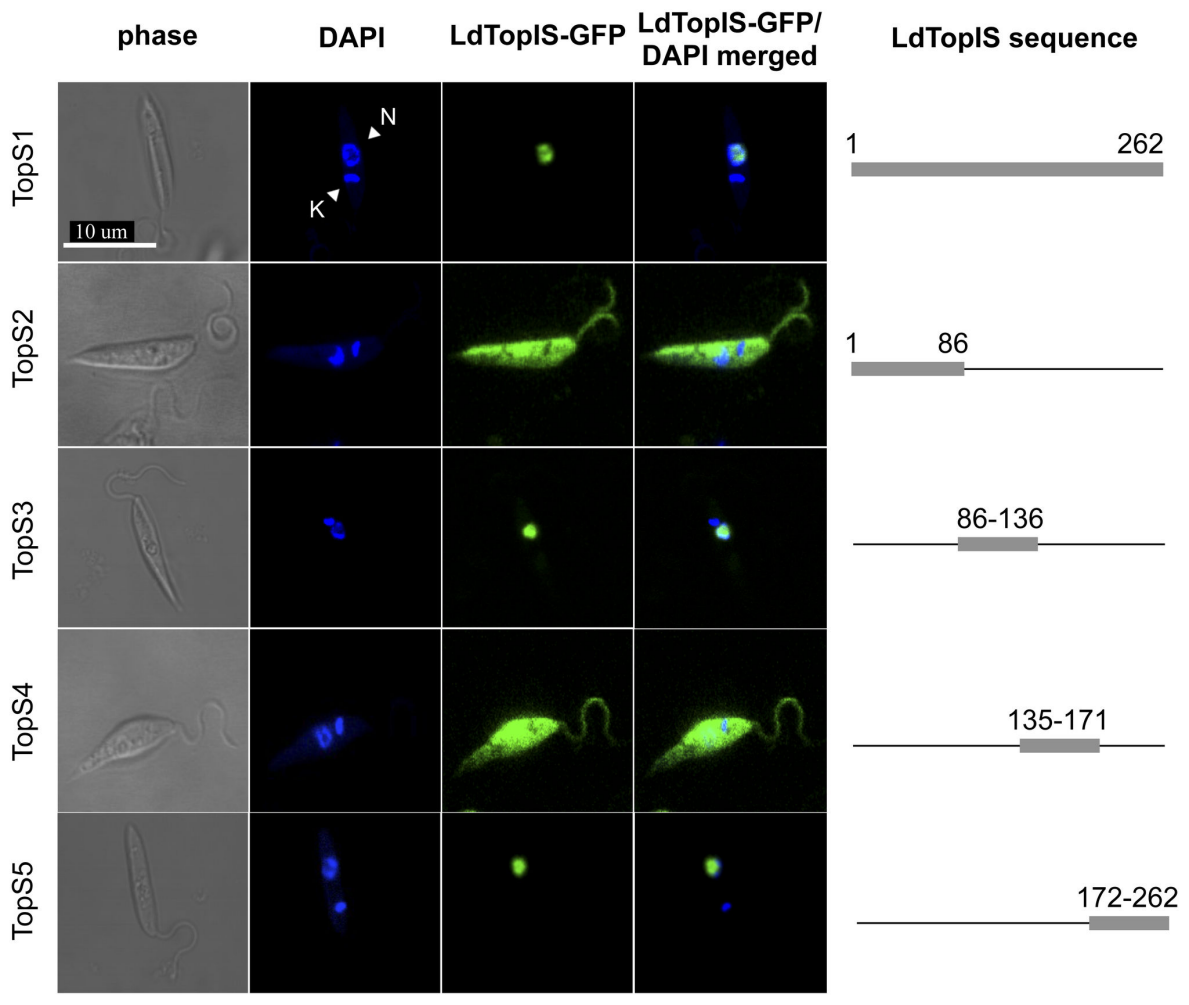
Microscopy confocal analysis of different fragments of the small LdTopIS subunit fused to GFP. Nucleus (N); kDNA (K). Genomic and kDNA staining was carried out with DAPI.

As no NLS motifs were expected to be present in LdTopIS it was amazing to find out how the entire small subunit fused to the GFP (TopS 1 aa 1-262), targeting the nucleus with no cytoplasmatic compartmentalization (Figure 3). This outcome prompted us to create new structures to define the location and extent of the motif/s responsible for nuclear transport of the protein.

LdTopIS N-terminal extension (aa 1-210) contains a long non-conserved motif (aa 85-136) rich in serine residues as well as an undecapeptide that is highly conserved in trypanosomatids (aa 171-183), which in turn, confers sensitivity to CPT [11]. Finally, the C-terminal end (aa 211-262) is phylogenetically conserved and contains the active site "SKxxY" in which Tyr-222 fulfils the function of cleaving the DNA substrate. We have also identified the similarity between the acidic-NLS DEDDAD motif (aa 117-146) described in the hTopIB [26] and DDDSSSDD sequence (aa 110 and 117) of LdTopIS. In fact, the APPPKVRRVE sequence (aa 86 and 96) is the only basic region within the N-terminal extension of the protein.

In the first extent four constructs were designed and fused to a GFP encoding gene in the pXG-GFP+2 at the end of the GFP ORF (Figure 3): TopS2 (aa 1-86), TopS3 (aa 86-136), TopS4 (aa 135-171) and TopS5 (aa 171-262).

TopS2 chimera amplified a fragment ending just before the start of the APPPKVRRVE sequence (aa 86-96). This fragment displayed a clear accumulation of green fluorescence in the cytoplasm (Figure 3) demonstrating the absence of functional NLS in the region. Promastigotes transfected with the TopS3 construct, which includes both APPPKVRRVE (aa 86-96) and DDDSSSDD (aa 110-117) motifs, exhibited nuclear localization, and consequently, a putative NLS is present within this LdTopIS partial sequence. These results confirm the presence of a NLS but do not exclude other regions being involved in the nuclear transport of the protein. TopS4 chimera comprises of the amino acids ranging in positions from 136 to 171, finishing just before the start of the undecapeptide PPRPPVVRSFE motif (aa 171-183), which plays role in CPT poisoning and is well conserved among trypanosomatids. This chimera showed cytoplasmic accumulation when microscopic analyses were carried out. Finally, in order to complete this first approach of LdTopIS dissection, TopS5 was designed. This construct contained the C-terminal fragment of the protein that remained unchecked (aa 171-262), which included the conserved catalytic domain (aa 211-262). Unexpectedly, fluorescence due to TopS5 was unequivocally accumulated in the promastigote nucleus (Figure 3). As a result, at this point we had identified two regions with the ability to internalize TopIS into the cell nucleus, corresponding to chimeras TopS3 and TopS5, respectively.

**Figure 4:**
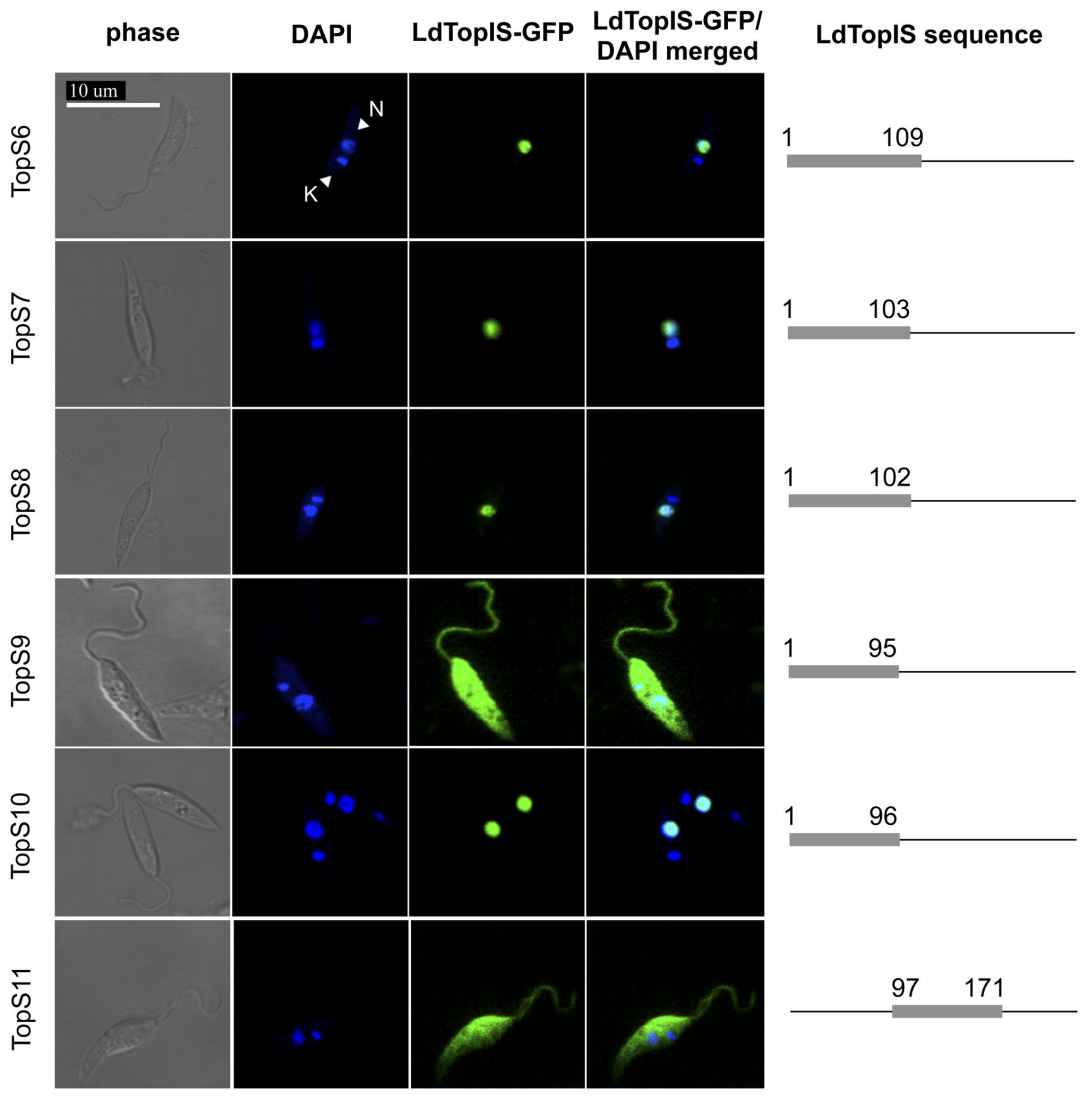
Deletion study of different length fragments of LdTopIS subunit fused to GFP by confocal microscopy. Nucleus (N); kDNA (K). Genomic and kDNA staining was carried out with DAPI.

TopS6 construct extends from the initial methionine to residue 109, being the sum of TopS2 chimera, the PPPKVRRVE motif and the entire serine cluster. Figure 4 shows that fluorescence due to TopS6 was accumulated in the cell nucleus, highlighting the importance of this signature motif for nuclear translocation. Between amino acids 96 and 109, in addition to the serine cluster, the only charged residue is Asp-103. To determine its possible role in the nuclear transport of LdTopIS, two new constructs were made. TopS7 construct includes the sequence extending from the initial methionine to Asp-103. On the other hand, TopS8 lacks Asp-103 ending at Ser-102 residue. Both chimeras were internalized in the nucleus, and as a consequence Asp-103 is not a main character in nuclear signalling. This implies a more exact delimitation of this NLS named NLS2 responsible for LdTopIS translocation, which must be located between amino acids 86 and 103.

In order to narrow the sequence constituting NLS2, a construct ending just before the serine cluster (TopS9) was cloned and put under study. As shown in Figure 4 the recombinant protein was unambiguously spread through the cytoplasm. Therefore, serine residues must be likely be involved in the translocation process, as PPPKVRRVE motif by itself is not able to achieve this function. Several chimeras containing this motif and increasing number of serine residues were generated (data non shown), but in the same way as was described for TopS10, the presence of a single serine residue completes the NLS and drives LdTopIS to the nucleus. This means that PPPKVRRVES, a ten-amino-acid basic sequence (aa 88-97), constitutes NLS2 in LdTopIS.

Despite having identified a NLS signature within this region, it still remained unsure the possible role of DDDSSSDD sequence (aa 110-117), which shares a high resemblance to the acid signal described for hTopIB (aa 117-146) [26]. To this end, we designed a new chimera involving amino acids 97 to 171 (TopS11). Fluorescence accumulation of the promastigotes transfected with this construct was unambiguously located in the cytoplasm. Therefore, the DDDSSSDD acid motif (aa 110-117) is not needed for the cytoplasm-nucleus transport.

**Figure 5:**
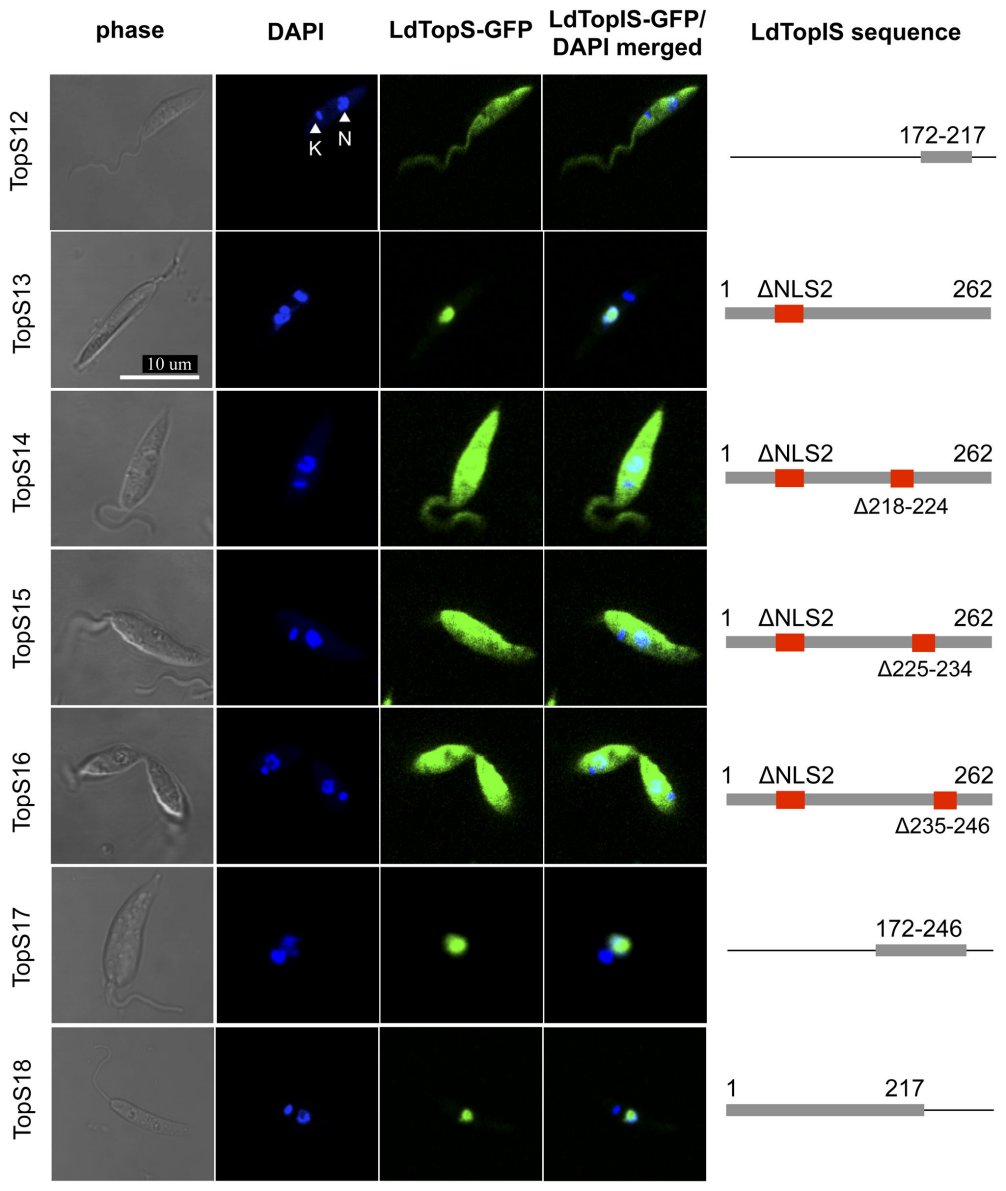
Identification of two NLS (red) motifs in LdTopIS subunit fused to GFP, by confocal microscopy. Nucleus (N); kDNA (K). Genomic and kDNA staining was carried out with DAPI.

Once NLS2 within the TopS3 sequence was defined, it was time to study the NLS identified region at the C-terminal end of the subunit. For this purpose we generated a TopS12 construct (Figure 5), which extends from residue 172 to 217 and includes the CPT-sensitivity-motif PPRPPVVRSFE [11]. This chimera was designed to end just before the SKINY catalytic site. Row 1 of Figure 5 shows that no nuclear fluorescence accumulation was achieved, pointing to the fact that NLS3 should be located between amino acids 218 and 262. To verify that the protein could be transported to the nucleus in the absence of NLS2, TopS13 chimera was generated. Figure 5 shows that the resulting chimera (row 2) is clearly driven into the nucleus in the absence of NLS2. By using TopS13 as the source, we decided to perform three serial internal truncations involving the region expected to include NLS3. These double-deleted chimeras were designed as follow: TopS14 for ΔNLS2/Δ218-224, TopS15 for ΔNLS2/Δ225-234 and TopS16 for ΔNLS2/Δ235-246. Rows 3 to 5 of Figure 5 show that the fluorescence due to all these constructs is dispersed through the cytoplasm, thus suggesting that the entire region could behave as a NLS. TopS17 (aa 219-246) was planned to assess if this region is playing the role of NLS3 within LdTopIS. Microscopy analysis revealed that this 28-amino-acids region drives the protein to the nucleus, thus pointing to a single and indivisible NLS (NLS3; aa 219-246) in LdTopIS.

Finally, we wanted to verify if the whole subunit could be transported to the nucleus in the absence of NLS3. TopS18 chimera along with TopS13 confirms that both NLS are able to drive LdTopIS to the cell nucleus independently.

### Both subunits can be transported independently to the nucleus

**Figure 6:**
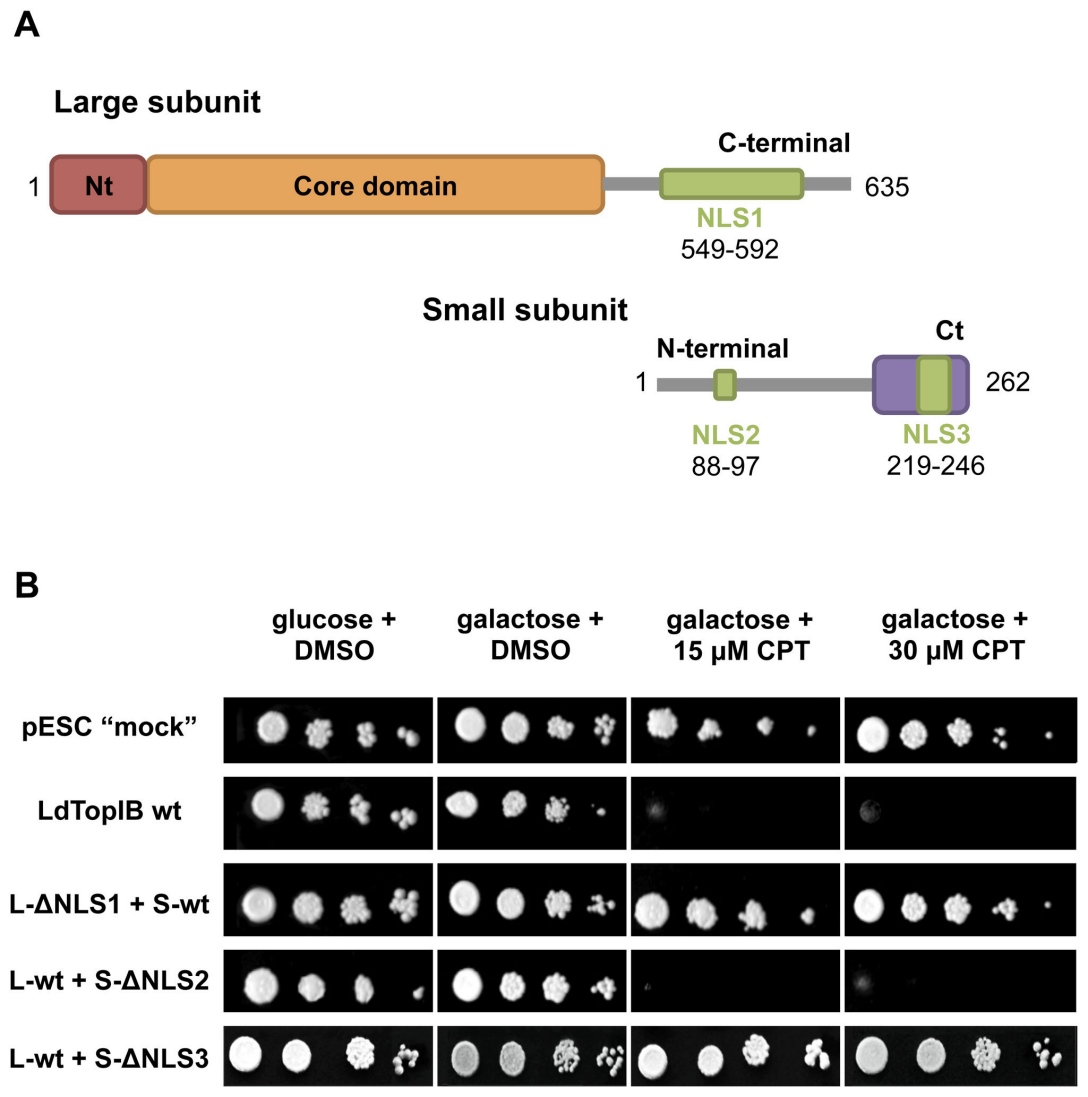
NLS3 is needed for nuclear transportation of LdTopIB. Spot tests show the sensitivity to CPT of MBY3 yeast strain transformed with the “empty” pESC-URA vector (“mock”) or carrying the wild type LdTopIB (wt) or different constructs lacking NLS1, NLS2 and NLS3, respectively. Pictures are representatives of multiple experiments.

Once NLS sequences were delineated in both LdTopIB subunits (Figure 6A), we decided to investigate whether these subunits are transported to the nucleus either jointly or independently. For this purpose we took advantage of the fact that LdTopIB is sensitive to CPT only when both subunits are assembled together inside the cell nucleus. TopIB-deficient MBY3 yeast strain was transformed with the wild-type protein (wt), the pESC-URA vector with no insert ("mock") and NLS-deficient LdTopIB chimeras (∆NLS). The resulting yeast cell viability assays (spot test) are shown in Figure 6B.

Because we have used a TopIB-deficient strain, the heterologous co-expression of both LdTopIB subunits would make MBY3 cells sensitive to CPT, thus inducing yeast death. As expected, all the constructs perfectly grew in the presence of glucose, because LdTopIB is only expressed in the presence of galactose (since both subunits were cloned under the control of GAL1/GAL10 promoters, respectively). In order to assess whether enzyme expression is deleterious or not by itself, cultures were grown in SC-Ura plates containing 2% (w/v) galactose and 0.5% (v/v) DMSO (CPT solvent). As shown in the second column of Figure 6B the growth of the transfected cultures is independent of LdTopIB expression in the absence of CPT. However, significant differences appeared when cultures where grown in the presence of the TopIB poison. Wild-type transfected cultures were unable to grow in the presence neither 15 µM nor 30 µM CPT, while the "mock" chimera showed no evidence of cellular damage at any tested concentration.

When NLS1 complex was removed from the large subunit (L-ΔNLS1 + S-wt) the culture displayed no sensitivity to any CPT concentration. As a result that no activity related residues were altered or removed [27], this indicates that the large subunit is unable to enter the nucleus by itself. Furthermore, the small subunit does not seem to be able to drag the whole heterodimer into the nucleus. When NLS2 was removed from LdTopIS (L-wt + S-ΔNLS2), cultures presented the same sensitivity profile as wild-type transfected ones. This indicates the presence of a functional TopIB within the yeast nucleus. In contrast, the culture expressing NLS3-lacking construction (L-wt + S-ΔNLS3) did not display sensitivity to CPT at all. We must remember that the catalytic Tyr-222 residue was removed within the NLS3 so the absence of sensitivity should be attributed to a non-functional enzyme, as it is unable to cut the DNA strand. These results all together indicate that both subunits could be transported to the nucleus independently. Besides, it demonstrates that, despite NLS2 being a totally functional signal, it is completely dispensable for the nuclear translocation of the small subunit *in vivo*.

### LdTopIB is not targeted to kinetoplast

To assess the subcellular localization of all the studied GFP-fusion proteins in *L. donovani* promastigotes, DNA was stained by incubation with a DAPI solution (see section 2.6.). As shown in all the figures in the present report, neither LdTopIL, nor LdTopIS fused chimeras were driven to the mitochondria and/or kDNA. These results demonstrate that LdTopIB lacks signature motives responsible for kinetoplast signaling or mitochondrial accumulation, which have been described in higher organisms.

## Discussion

To catalyze changes in DNA superhelicity, TopIB should be located inside the nucleus, where it produces temporary single DNA-chain scissions, which allows relaxation before the encoded information is accessible for transcription, reparation or replication enzymes [28].

Proteins operating in the nucleus are synthesized in cytosol and therefore, they must be driven to the nucleus by a specific group of karyopherins called importins. Importins translocate those proteins that contain recognizable NLS motifs in their sequence [29–31]. NLS sequences are highly conserved among higher eukaryotes, nevertheless, there are very few studies describing functional NLS in trypanosomatids [19–22]. As a consequence, it has not been possible to find a common sequence responsible for nuclear transport in these parasites.

Both its nature and length are very heterogeneous factors in trypanosomatids NLS. While Kumar et al. [19] found a sequence of four amino acids in the highly basic N-terminal end of the ORC1 protein; other research groups have found long amino-acid sequences where NLS are dispersed inside. In this report we have identified three NLS motifs in LdTopIB – one in the large subunit and two in the small one –by partial deletions of the genes encoding both subunits fused to pXG-GFP+2. NLS1 is located in the C-terminal extension of LdTopIL and consists of a basic sequence of 44 amino acids where 18 lysine residues, which are scattered throughout the region. This non-canonical distribution of NLS similarly resembles one found for *L. major* KIN13-1 kinesin, a 60-residues-length non-canonical NLS placed at the C-terminal end of the protein [22]. Another study outlined a 60-amino acids NLS region in the C-terminal extension of *L. donovani* Tdp1 [20]. A deletion study of the gene encoding *L. donovani* Top II fused to GFP delimited another region of around 60 amino acids at the C-terminal end, which contains potential NLS motifs responsible for the translocation of this enzyme to the cell nucleus [21]. These results confirm the difficulty of defining amino-acid sequences responsible for nuclear targeting in *Leishmania* [20,21].

NLS2 is a ten-residues-length motif placed at the N-terminal end of LdTopIS, heading a large cluster of serine residues. This fact is relevant, as the PPPKVRRVES peptide requires the presence of at least one Ser residue to fully complete a functional NLS. A plausible explanation could be that at least one Ser residue must be phosphorylated for transportation to take place, as previously described for other NLS motifs within different proteins [32]. In the end, NLS3 was identified in the region that includes the catalytic Tyr-222 within the C-terminal end of LdTopIS. This signal has no resemblance to other canonical NLS signals described so far, however, it may bear a resemblance, both structurally and in sequence, to the PY-NLS signals proposed by Lee et al. [33]. According to this model, importins might be binding to a Pro closely spaced to a Tyr residue of the target protein, thus producing the nuclear translocation of the peptide. LdTopIB NLS3 includes the sequence of 218-SKINYIDPR-226, where Tyr-222 and Pro-225 are found close together [34].

Some research groups have hypothesized that LdTopIB must be necessarily assembled in the cytoplasm to be translocated to the cell nucleus. Furthermore, LdTopIL should be acting as a carrier of the proenzyme [16]. However, our results suggest that both subunits could reach the nucleus independently as there is at least one functional NLS motif in each monomer. Furthermore, it has been recently demonstrated that NLS1 forms part of the interaction region of the large monomer with the LdTopIS [11], preventing the binding to importins. Therefore, if both proteins are independently folded and assembled in the cytoplasm, the responsibility of nuclear transport should fall on NLS3, as spot tests showed that NLS2 is completely dispensable for nuclear translocation *in vivo*.

There are several studies that show that eukaryotic TopIB is dually accumulated in both nucleus and mitochondria [13]. Trypanosomatids are primitive eukaryotes with an early genetic divergence. These parasites have a single modified mitochondrion that houses a single organelle adjacent to the flagellar basal body, called a kinetoplast. This organelle contains an intricate network of kDNA organized in both mini and maxicircles, whose replication occurs simultaneously in the nuclear DNA during cell division [14]. Shapiro et al. [15] have shown that TopII and TopIA are placed inside both the nucleus and the single mitochondrion of *T. brucei*, both associated with DNA minicircles and therefore, they are operative to solve any topological problem within the organelles during cell replication. However, Das and coworkers [35] indicated that LdTopIB co-localizes inside the kinetoplast associated with DNA minicircles. To verify these results, we conducted a cell DNA staining with DAPI in transfected promastigotes of each LdTopIB-GFP constructs. Our results clearly showed that GFP was not accumulated in the kinetoplast at all, thus proving that none of LdTopIB subunits are bound to DNA minicircles, which in fact rules out the hypothesis of a dual localization of the enzyme.

## Conclusions

Three putative NLS sequences have been identified in LdTopIB. LdTopIL subunit contains the NLS1 motif formed by 44 amino acids located in its C-terminal end. This is a large region within which small groups of adjacent basic amino acids can assume the role of driving the protein to the cell nucleus. LdTopIS subunit contains two NLS. NLS2 sequence is comprised of a PPPKVRRVES motif located between positions 88 and 97, whereas NLS3 involves 28 amino acids (residues 219-246) at the C-terminal end including the catalytic Tyr-222. Finally, neither of the entire subunits, nor any of the constructs fused to GFP were driven to the kinetoplast.
